# The Relation of Diet to Personal Beauty

**Published:** 1891-02

**Authors:** John V. Shoemaker

**Affiliations:** Professor of Therapeutics and Materia Medica, Dermatology and Clinical Medicine in the Medico-Chirurgical College of Philadelphia; Author of a Treatise on Skin Diseases; the Oleates in Skin Diseases; Health and Personal Beauty, &c., &c.


### The Relation of Diet to Personal Beauty.

BY JOHN V. SHOEMAKER, A M., MD.

Professor of Therapeutics and Materia Medica, Dermatology and Clinical Medicine in the Medico-Chirurgical College of Philadelphia; Author of a Treatise on Skin Diseases; the Oleates in Skin Diseases; Health and Personal Beauty, &c., &c.

Thomas Carlyle, a mighty thinker, but a man of perverse moods and a chronic dyspeptic, wrote admiringly of an ideal "eupeptic" man as one who was brave, dutiful and dominant. From the depths of his unhappy experience, a victim to "the vile hag, Dyspepsia," the rugged Scotchman perceived the immense advantage which (other things being equal) the man of good digestion possesses in the battle of life. Carlyle's hero is emphatically a strong man; but viewing him from another standpoint we may also be permitted to affirm that he is a handsome man. This statement may seem to be made in the spirit of the time-honored adage, "handsome is that handsome does." It admits, however, of a ready explanation. For in what does personal beauty consist? What are its chief elements? We are not able, it may be said, to add to, or take from our stature; we can not alter the contour of head and face, and can not train features to beauty of form. These objections are perfectly valid. At this period of the world's history, after the great fusion of races, or at least, tribes which has taken place in Europe, we can not expect to find the ideal purity of feature which distinguished the nations of classical antiquity, and especially the Greeks. Perfect regularity of features, or facial beauty, from an absolutely artistic point of view, we do not look for, scarcely even desire in men. That which adorns man is the stamp of intellect and strength set upon his face. Beauty in the male chiefly consists in a well-developed form, and an expressive countenance. Functionally active facial muscles perpetually responding to the impulses of an active mind give interest, variety, and in a sense, beauty, to features otherwise of an ordinary type. That intellectual pursuits bestow dignity and distinction upon the human countenance is a fact noticed in an Egyptian poem of the XIIth Dynasty, written perhaps 2,000 years B. C.

But when we speak of personal beauty it is especially of woman that we think. In woman a rounded outline, grace of movement, delicacy and regularity of features, fairness of skin, gloss and luxuriance of hair, carry beauty to perfection. Yet, even in the "fair sex" *par excellence*, the charmed beholder seldom pauses, or cares to pause, to criticise the form of features animated with ever-varying expression. Our highest admiration is not given to the

* * * * "cold and clear-cut face, as I found when her carriage past,
Perfectly beautiful: let it be granted her: where is the fault?
All that I saw (for her eyes were downcast, not to be seen)
Faultily faultless, icily regular, splendidly null,
Dead perfection, no more;"

but to the face instinct with love, mirth, and vivacity. Beauty resides in the pure skin, sparkling eye, healthy bloom, pearly teeth, ripe and smiling lips, a refined, mobile, and intellectual expression. And these are not fortuitous possessions. Disregarding, for the present, the influence of heredity we may confidently assert that the essential attributes of beauty depend, to a great extent, upon proper food, or more strictly, upon proper digestion. For, as I have elsewhere pointed out,* food, though abundant and nutritions, may be so poorly prepared that the appetite is not excited, the palate pleased, or digestion thoroughly performed.

Perfect development depends upon perfect nutrition. It is certain that nutrition can not be perfect unless the digestive apparatus properly performs its functions. If the normal pro-

* Heredity, Health and Personal Beauty, by John V. Shoemaker, A.M., M.D., Philadelphia and London; F. A. Davis, 1890.

portion of acid and ferment be disturbed in the gastric juice, or if the liver, pancreas, or intestinal glands are deranged, if inactive kidneys and constipated bowels check the elimination of deleterious bye-products, the skin loses its softness and the complexion becomes dull. The beautiful violet tinge of the sclerotic gives place to a muddy hue, the flashing eye grows dull and heavy. The cellulo-fatty tissue of the face wastes, the features grow thin, and wrinkles appear. Acne, seborrhœa, eczema, urticaria, erythema, or furuncles may develop upon the face, the hair is liable to become dull and thin, and the teeth to decay. The angles of the mouth droop, and a haggard, anxious glance replaces the frank aspect of health. Irregularity of features which was scarcely noticed when the eye was bright and sparkling, the skin pure, and the complexion brilliant, now becomes painfully apparent.

It is doubtless true that woman is not to be prized solely for her beauty. Nevertheless, her beauty must always be a magnetic charm and is justly regarded by herself as a valued possession. Our American women are apt to fade easily because they neglect the laws of health. Women as well as men participate in, and are injured by the keen competition and ambitious struggles of our social and commercial life. Among the wealthy the whirl of society, late hours, rich food, and neglect of physical exercise, breed indigestion and its attendant ills, and art is called upon in vain to repair or cancel ravages due to defiance of natural laws. In the poor or the middle classes coarse, insufficient, or badly cooked food, eaten hastily, without appetite and with an anxious, pre-occupied mind, transform the pretty bride, all too soon, into the thin, plain, jaded mother and household drudge. Good food, well-prepared, eaten leisurely and properly digested, preserves cheerfulness, a pleasant interest in affairs of life, health, and what is by no means least deserving of our consideration, personal beauty.—*Dietetic Gazette.*

